# Repurposing MK0873 as a non-covalent hTERT-Targeting compound: a proof of concept study

**DOI:** 10.3389/fragi.2026.1783019

**Published:** 2026-08-26

**Authors:** Maham Ansari, Abdul Wali, Mohhamed Rasul, Kristina Gemzell Danielsson, Twana Alkasalias, Muhammad Mushtaq

**Affiliations:** 1 Department of Biotechnology, Faculty of Life Sciences and Informatics, Balochistan University of Information Technology, Engineering and Management Sciences (BUITEMS), Quetta, Pakistan; 2 Department of Women’s and Children’s Health Karolinska Institutet, Stockholm, Sweden; 3 Department of Pharmaceutical Basic Science, Faculty of Pharmacy, Tishk International University, Erbil, Iraq; 4 Department of Gynecology and Reproductive Medicine, Karolinska University Hospital, Stockholm, Sweden; 5 General Directorate of Scientific Research Center, Salahaddin University-Erbil, Erbil, Iraq; 6 Department of Microbiology Tumor and Cell Biology, Karolinska Institutet, Stockholm, Sweden

**Keywords:** cellular senescence, drug repurposing, MK0873, neuroblastoma, telomerase inhibition

## Abstract

Telomerase is a key regulator of both cellular immortality and aging, making it an attractive target for cancer therapy and age associated pathologies. Its clinical exploitation has been limited by structural complexity and challenges in achieving specificity. Recent structural insights into human telomerase reverse transcriptase (hTERT) have enabled the identification of selective inhibitors. Here, we repurposed MK0873, a PDE4 inhibitor previously evaluated in Phase II clinical trials for rheumatoid arthritis and COPD, as a non covalent telomerase targeting agent. *In silico* docking and molecular dynamics simulations predicted stable binding of MK0873 to hTERT, distinct from the covalent interaction of BIBR1532. Analysis of neuroblastoma datasets revealed that high TERT expression correlates with poorer survival, independent of MYCN amplification. MK0873 reduced proliferation of hTERT positive cells, promoted cellular senescence and decreased relative telomere length, whereas ALT positive cells exhibited minimal response, supporting selective activity in telomerase dependent cellular models. Importantly, direct biochemical assessment using the Telomeric Repeat Amplification Protocol assay demonstrated a significant reduction in telomerase activity following MK0873 treatment, providing experimental evidence that complements the computational predictions and cellular phenotypes observed in this study. Although the magnitude of telomerase inhibition was lower than that of the reference inhibitor BIBR1532 under identical experimental conditions, the combined computational, biochemical and cellular findings consistently support hTERT as a relevant molecular target of MK0873. Collectively, these results provide proof of concept evidence that MK0873 represents a promising lead scaffold for non covalent targeting of hTERT. While additional studies are required to further define cellular target engagement, distinguish potential PDE4 dependent effects and optimize inhibitory potency. Current work establishes a foundation for the continued development of MK0873 derived compounds as potential telomerase targeted anticancer therapeutics.

## Graphical Abstract

**Figure:**
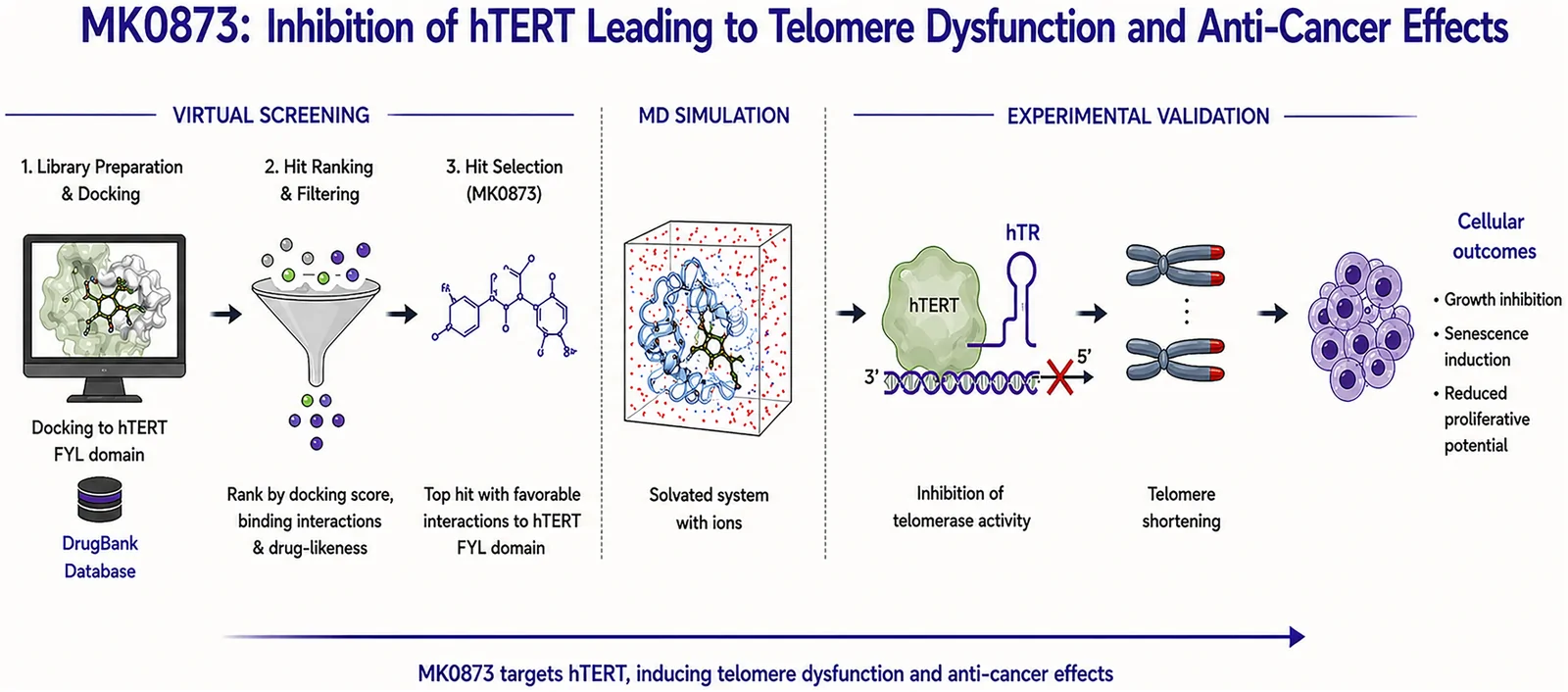
Graphical Abstract: A structure-guided drug repurposing workflow identified MK0873 from the DrugBank database as a novel inhibitor targeting the hTERT FYL domain. Following virtual screening and molecular dynamics simulations to validate stable binding, experimental studies confirmed telomerase inhibition by MK0873. Sustained inhibition led to telomere shortening, reduced proliferative capacity, and induction of cellular senescence.

## 1 Introduction

Human telomerase reverse transcriptase (hTERT) is the catalytic subunit of the telomerase that promotes the limitless replicative proliferation via telomere length maintenance, a significant hallmark of the cancer (Hanahan and Weinberg, 2011). In normal somatic cells, the expression of hTERT is tightly regulated (Chen et al., 2020), whereas it is aberrantly reactivated in 85%–90% of the malignancies (Akincilar et al., 2016; Kim et al., 1994; Doğan et al., 2019). About 10%–15% of the cancer cases report the alternative lengthening of telomerase (ALT) (Amato et al., 2020).

The universal expression of hTERT across cancers makes it an interesting target for the development of anticancer therapies. The telomeres of the malignant cells are relatively shorter ∼5 kb compared to the ∼10–20 kb length in stem cells and normal somatic cells (Kelland, 2005; Phatak and Burger, 2007). Therefor targeting hTERT will infer selective cytotoxicity on malignant cells and minimal risk to normal cells (Corey, 2000). hTERT targeted therapies can reduce the tumor burden by promoting telomere shortening in rapidly dividing malignant cells and activates the cascade of cellular senescence (Bryan and Reddel, 1997; Holysz et al., 2013; Lingner et al., 1997; Shay and Bacchetti, 1997).

BIBR1532 is one of the prominent small molecule inhibitor, that directly inhibits the activity of the telomerase leading to the progressive telomere shortening, triggering cell senescence and limiting cell proliferation (Doğan et al., 2019; Shi et al., 2015; Kong et al., 2015; El Daly and Martens, 2007). BIBR1532 binds to the FVYL motif consisting of conserved hydrophobic residues (Phe1012, Val1025, Tyr1089 and Leu1092) in hTERT. This hydrophobic pocket is located at the outer surface of thumb domain adjacent to the TRBD domain and provides stability to the surrounding alpha helices and loops (Bryan et al., 2015).

Despite the extensive research, direct pharmacological inhibition of hTERT has remained challenging because of its structural complexity and adaptive drug resistance (Jafri et al., 2016; El-Daly et al., 2005). Current hTERT targeting strategies demonstrate poor pharmacokinetics with limited efficacy (Harley, 2008; Shay and Keith, 2008; Shay and Wright, 2002). In this paradigm, small molecules signify a critical niche. These are low molecular weight compounds that can diagnose, treat and prevent disease by tempering biochemical processes. These compounds can modulate protein function by binding to hydrophobic pockets, mimicking the role of the endogenous substrate. Small molecule drugs have remained an attractive target given their simple chemical structure and predictable pharmokinetics and pharmacodynamics, high stability and simple dosing protocols (Ngo and Garneau-Tsodikova, 2018).

Launching a new candidate into the drug market is very challenging with the slow pace, high attrition rate and development cost. In this context, drug repurposing offers an alter route to identify the new therapeutic uses of the existing or investigational drugs for both common and rare diseases. Compared to *de novo* drug discovery, leveraging molecules with the known biosafety profile, manufacturing and bioavailability data, drug repurposing can reduce the attrition rate and can shorten the drug development timeline (Pushpakom et al., 2019; Ashburn and Thor, 2004).

Phosphodiesterase (PDE) inhibitors have recently attracted increasing attention as potential anticancer agents because cyclic nucleotide signaling regulates cell proliferation, apoptosis, inflammation, angiogenesis and immune responses within the tumor microenvironment. While PDE5 inhibitors have been investigated most extensively, emerging evidence suggests that inhibition of other PDE family members, including PDE4, may also exert antitumor effects in selected malignancies (Nemr et al., 2025; Nemr et al., 2023). MK0873 was originally developed as a selective PDE4 inhibitor and advanced to Phase II clinical evaluation for inflammatory diseases, demonstrating a favorable pharmacokinetic and safety profile. In the present study, however, we investigate MK0873 from a different perspective, evaluating its potential as a direct hTERT targeting small molecule identified through structure based virtual screening.

Nevertheless, telomerase has remained an important yet challenging target in oncology, that can selectively exhaust the cancer cells without compromising the normal cells. Yet the efforts done so far has produced no effective hTERT inhibitor that provides long term inhibition with clinical viability and target specificity. Our research aims to address the scarcity of the potential hTERT inhibitor. In this study, we employed a structure based drug repurposing strategy to identify clinically characterized compounds capable of targeting the conserved FVYL motif of hTERT. Computational screening identified MK0873 as a promising candidate, which was subsequently evaluated using molecular docking, molecular dynamics simulations, TRAP assay, telomere length analysis, proliferation assays and senescence assays. Our objective was to assess whether MK0873 could serve as a proof of concept non covalent hTERT targeting scaffold for further development.

## 2 Materials and methods

### 2.1 Virtual screening

The DrugBank database (Knox et al., 2024) (9,468 compounds) was virtually screened against the FVYL binding pocket of human telomerase reverse transcriptase (hTERT; PDB ID: 7BG9) using AutoDock Vina. The top ranked compounds were selected based on docking score for subsequent MM/GBSA rescoring. Detailed ligand preparation, receptor preparation, docking parameters and virtual screening workflow are described in Supplementary Methods S1.

### 2.2 Binding free energy calculations using molecular mechanics/generalized born surface area (MM/GBSA)

The top 200 compounds were rescored using the Uni-GBSA workflow to estimate binding free energies and ligand efficiencies. Detailed MM/GBSA calculation procedures are provided in Supplementary Methods S2.

### 2.3 Redocking using GOLD

The MM/GBSA top ranked molecules were re-docked using GOLD (Genetic Optimization for Ligand Docking, Version 5.3.0), which allows partial protein flexibility around the active site through Genetic Algorithms (Jones et al., 1997). Top poses were evaluated based on visual inspection and molecules showing consistent orientations in both AutoDock Vina and GOLD were identified as lead candidates.

### 2.4 Molecular dynamics (MD) simulations using GROMACS

Molecular dynamics (MD) simulations of the lead complexes were performed using GROMACS 2023.1 (Abraham et al., 2015). MD was first run for 400 ns, followed by a second 100 ns run starting from the final frame of the first trajectory, resulting in a total of 500 ns simulation. Complete simulation parameters are described in Supplementary Methods S3.

### 2.5 Analysis of hTERT expression and survival in neuroblastoma patients

Publicly available neuroblastoma gene expression and clinical outcome data were obtained from the Kocak dataset (Kocak et al., 2013) using the R2 Genomics Analysis and Visualization Platform (https://r2.amc.nl). This dataset corresponds to the Gene Expression Omnibus accession number GSE45547 and includes primary neuroblastoma tumor samples with annotated MYCN amplification status and patient survival information. Tumors were stratified based on MYCN amplification and hTERT (TERT) expression levels, with patients categorized into high- and low-expression groups using the median expression value as a cutoff. Kaplan–Meier survival analyses were performed within R2 to assess the association between hTERT expression and overall survival. Statistical significance was determined using the log rank test.

### 2.6 Cell culture

Neuroblastoma cell lines IMR32 (ATCC CCL-127), SKNBE(2) (ATCC CRL-2271) and SK-N-SH (ATCC HTB-11) were selected based on their expression of hTERT as reported in (Lindner et al., 2015). These were cultured in a 1:1 mixture of Nutrient Mixture F12 (Sigma-Aldrich, #N6658) and Minimum Essential Medium Eagle (Sigma-Aldrich, #M4655), supplemented with 10% fetal bovine serum (FBS; Nordic Biolabs, #SV30160.03HI), 1% penicillin/streptomycin, 1% non-essential amino acids and 1% L-glutamine. NCIH358 (ATCC CRL-5807) cells were maintained in RPMI 1640 medium (Sigma-Aldrich, #R8758) with 10% FBS, 1% penicillin/streptomycin and 1% L-glutamine. hTERT-immortalized human fibroblast BjTERT cells (ATCC CRL 4001) were cultured in DMEM supplemented with 10% FBS, 1% penicillin/streptomycin and 1% L-glutamine. All cell lines were incubated at 37 °C in a humidified atmosphere containing 5% CO_2_.

### 2.7 Telomeric Repeat Amplification Protocol (TRAP) assay

Telomerase activity was determined using the TRAPEZE™ XL Telomerase Detection Kit (Sigma-Aldrich, Cat. No. S7707FR) according to the manufacturer’s instructions. Jurkat cells were lysed using the CHAPS lysis buffer provided with the kit and protein concentration was determined prior to analysis. For each reaction, 30 ng of total protein was added to a final reaction volume of 20 μL. MK0873 (20, 40, or 60 µM) (Cat. No. T33422, TargetMol, Boston, MA, United States), BIBR1532 (20 µM) (Cat. No. T2380, TargetMol, Boston, MA, United States) or an equivalent volume of DMSO was added directly to the TRAP reaction mixture to evaluate the direct effect of each compound on telomerase activity. PCR amplification was performed using Platinum™ Taq DNA Polymerase (Invitrogen). Following amplification, fluorescein fluorescence (ΔFL) was measured at an excitation/emission wavelength of 485/535 nm and sulforhodamine fluorescence (ΔFR) was measured at 585/620 nm using a fluorescence microplate reader (SpectraMax® iD3). Telomerase activity was calculated as the ΔFL/ΔFR ratio according to the manufacturer’s instructions. The ΔFL/ΔR ratio obtained from the negative control was subtracted from the corresponding ratio of each experimental sample and the resulting values were used for statistical analysis. Data are presented as mean ± SD from three independent biological experiments. Statistical significance was determined using one way ANOVA followed by Dunnett’s multiple comparisons test.

### 2.8 Telomere length measurement assay

Telomere length was measured following the protocol described in (Joglekar et al., 2020). All the cells were seeded at subconfluent density of 60,000 cells per well in 6 well plates and treated the next day with 10 μM of DMSO (Life Technologies), MK0873, or BIBR1532 for 4 days. Detailed method is provided in Supplementary Methods S4.

### 2.9 X-gal staining for SA-β-gal activity

BjTERT cells (5,000 cells/well) were seeded in 96 well plates in triplicate. The following day, cells were treated with DMSO, 5 μM, or 10 μM of BIBR1532 or MK0873 for 5 days. Cells were then washed twice with PBS and fixed with 0.2% glutaraldehyde. The X-gal staining solution contained 2 mM MgCl_2_, 5 mM potassium ferricyanide, 5 mM potassium ferrocyanide and 1 mg/mL X-gal. 100 μL of the solution was added per well and incubated overnight at 37 °C without CO_2_. X-gal positive cells were counted and data were analyzed and plotted using GraphPad Prism.

## 3 Results

### 3.1 *In silico* screening

The entire DrugBank library (9,468 molecules) was screened against the catalytic core of telomerase (hTERT), focusing on the FVYL motif using AutoDock Vina. Results were extracted using Python scripts as described (Shah et al., 2024), molecules were ranked based on binding affinity, with the top 200 compounds showing values from −8.6 to −6.0 kcal/mol (Supplementary Table S1).

These top hits were further evaluated using MM/GBSA to refine their binding affinity and distinguish strong binders from weaker ones. The analysis provided detailed interaction profiles, including polar and non polar solvation contributions. The top hit molecules exhibited ΔGbind values ranging from −50.8 to −43.5 kcal/mol and were also assessed for ligand efficiency (LE), ensuring that strong binding was balanced with molecular size (Supplementary Table S1).

**FIGURE 1:**
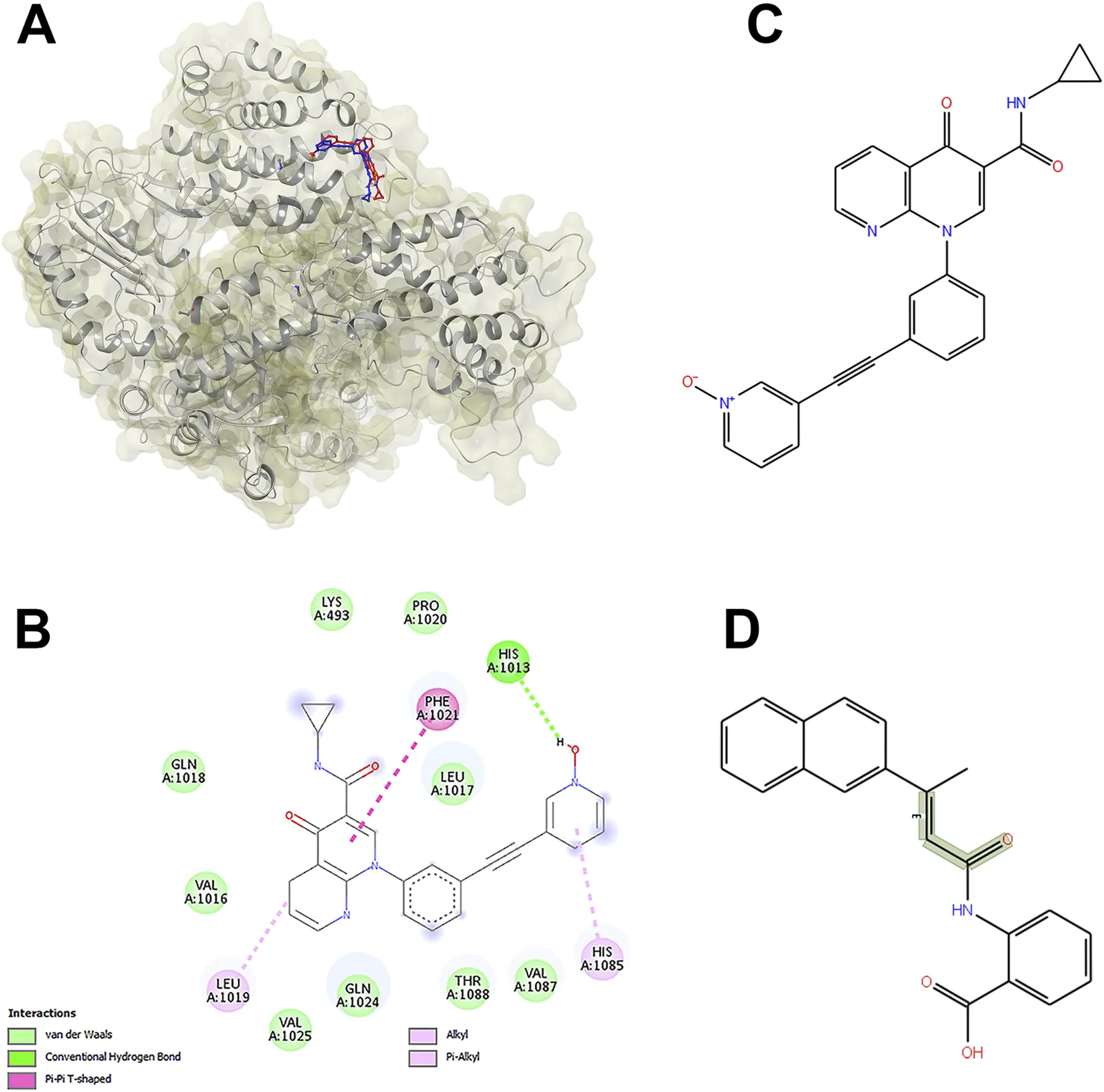
**(A)** Docking poses of MK0873 in the FVYL motif of hTERT, predicted by AutoDock Vina (blue) and GOLD (red), showing consistent orientation across both docking platforms. **(B)** Chemical structure of MK0873. **(C)** Detailed interactions of MK0873 with key residues in the FVYL domain, including conventional hydrogen bonds and non conventional contacts that stabilize the ligand within the binding pocket. **(D)** Chemical structure of BIBR, with the Michael acceptor group highlighted in green.

To identify the most promising leads, the top 50 compounds were re-docked using GOLD and poses were visually compared with AutoDock Vina derived poses. Only one molecule, DB13029 (MK0873), showed consistent docking orientation across both platforms Figure 1A. MK0873 established conventional hydrogen bonds and additional non conventional interactions Figure 1B, ensuring stable binding, optimal orientation within the binding pocket. Key residues of hTERT protein involved in interaction with MK0873 and BIBR1532 are given in Table 1.

**TABLE 1:** Key residues of hTERT protein involved in interaction with MK0873.

Compound	Residue	Interaction	Distance (Å)
MK0873	His1013	Hydrogen bond	2.70
	Phe1021	π–π stacking	—
	Leu1019	π–alkyl	—
BIBR1532	Phe1021	Hydrophobic/π interaction	—
	Gln1024	Polar contact	3.80

Comparison of binding metrics indicates that MK0873 interacts more favorably with hTERT than BIBR1532. Specifically, MK0873 showed a lower AutoDock Vina score (−7.3 vs. −6.9) and a more favorable MM/GBSA binding free energy (−41.26 vs. −37 kcal/mol). Chemical structure analysis of MK0873 (Figure 1C) and BIBR1532 (Figure 1D) revealed that BIBR1532 contains a potentially reactive chemical moiety, highlighted in green in Figure 1D, which may contribute to off target effects.

### 3.2 Analysis of receptor–ligand interactions via molecular dynamics simulation (GROMACS)

**FIGURE 2:**
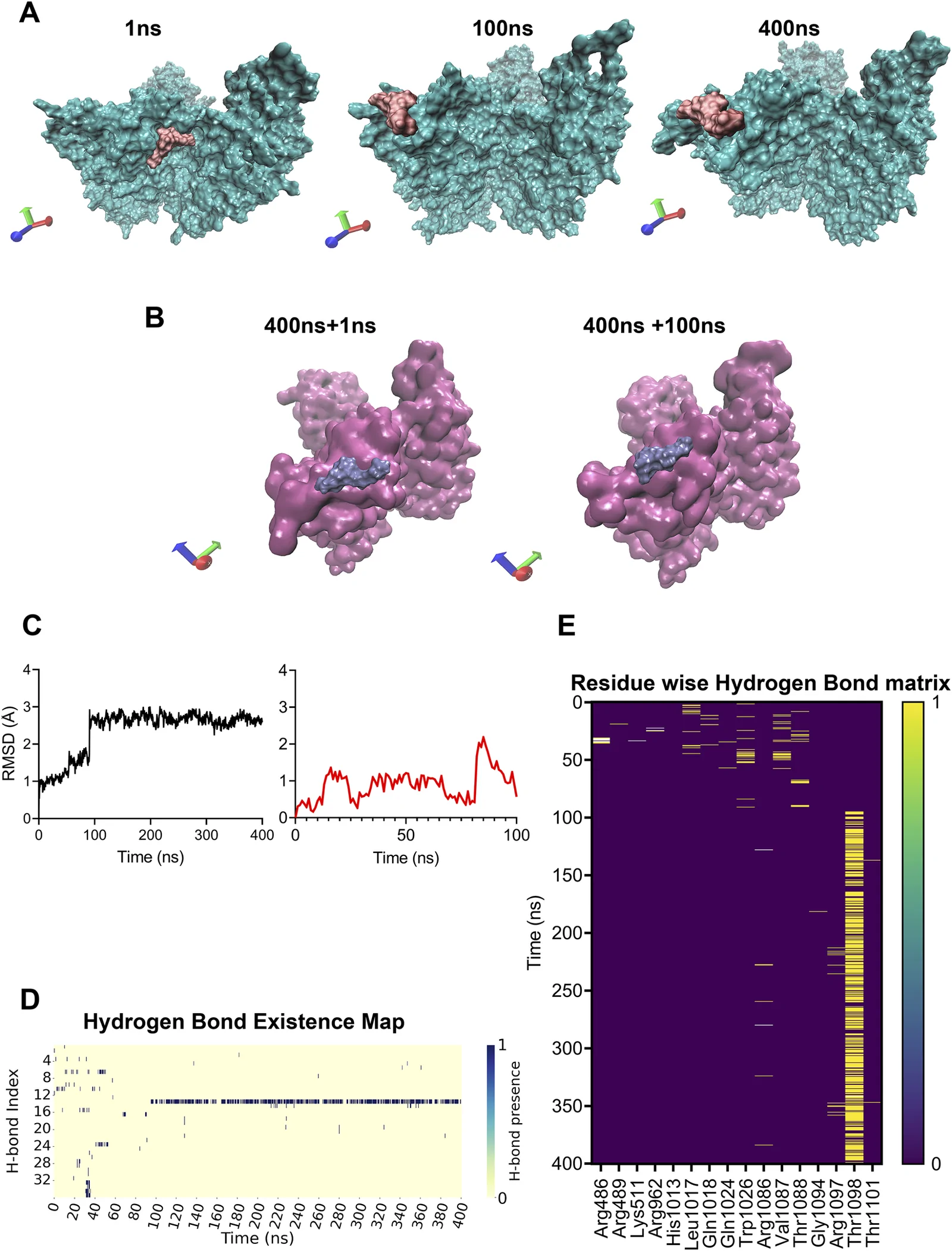
Molecular dynamics simulation of MK0873 bound to the FVYL domain of hTERT. **(A)** Surface representation of the MK0873–hTERT complex at 0 ns (left), 100 ns (middle) and 400 ns (right) during the first 400 ns simulation. **(B)** Surface structure of the extended simulation showing the complex at 400 ns + 0 ns (left) and 400 ns + 100 ns (right). **(C)** RMSD of MK0873 relative to hTERT showing stable fluctuations below 1 Å during 100–400 ns (left) and continued stability between 1 and 2 Å during the additional 100 ns simulation (right). **(D)** Hydrogen bond index of MK0873 with hTERT over the 400 ns simulation. **(E)** Key interacting residue Thr1098 maintains a stable hydrogen bond with MK0873 throughout the simulation.

To investigate the stability and interactions of MK0873 with hTERT, a 500 ns molecular dynamics simulation was performed in two stages. During the initial 400 ns simulation, MK0873 was initially bound to the FVYL domain of hTERT. Around 90 ns, the ligand shifted slightly within the binding pocket and by 100 ns it established a stable hydrogen bond with Thr1098 that persisted until the end of the simulation. Key residues involved in interactions with MK0873 included Arg1086, Val1087, Val1090, Pro1091, Leu1093, Gly1094, Arg1097, Thr1098 and Thr1101 (Figure 2A).

The complex was further simulated for an additional 100 ns starting from the final frame of the first trajectory. MK0873 remained bound in the same region throughout this extension, confirming the stability of the ligand–protein complex (Figure 2B).

RMSD analysis revealed that after initial fluctuations, the ligand achieved a stable conformation, with deviations remaining below 1 Å between 100 and 400 ns. In the subsequent 100 ns simulation, RMSD fluctuations remained low, ranging from 1 to 2 Å, indicating that MK0873 continued to maintain a stable interaction within the binding pocket (Figures 2C,D).

Hydrogen bond analysis over the first 400 ns confirmed that Thr1098 formed a consistent hydrogen bond with MK0873, while other residues such as Arg1086 and Arg1097 contributed intermittently (Figures 2D,E). Overall, these results demonstrate that MK0873 maintains a stable binding orientation within the FVYL pocket of hTERT over the 500 ns simulation period.

### 3.3 hTERT expression in neuroblastoma patients

**FIGURE 3:**
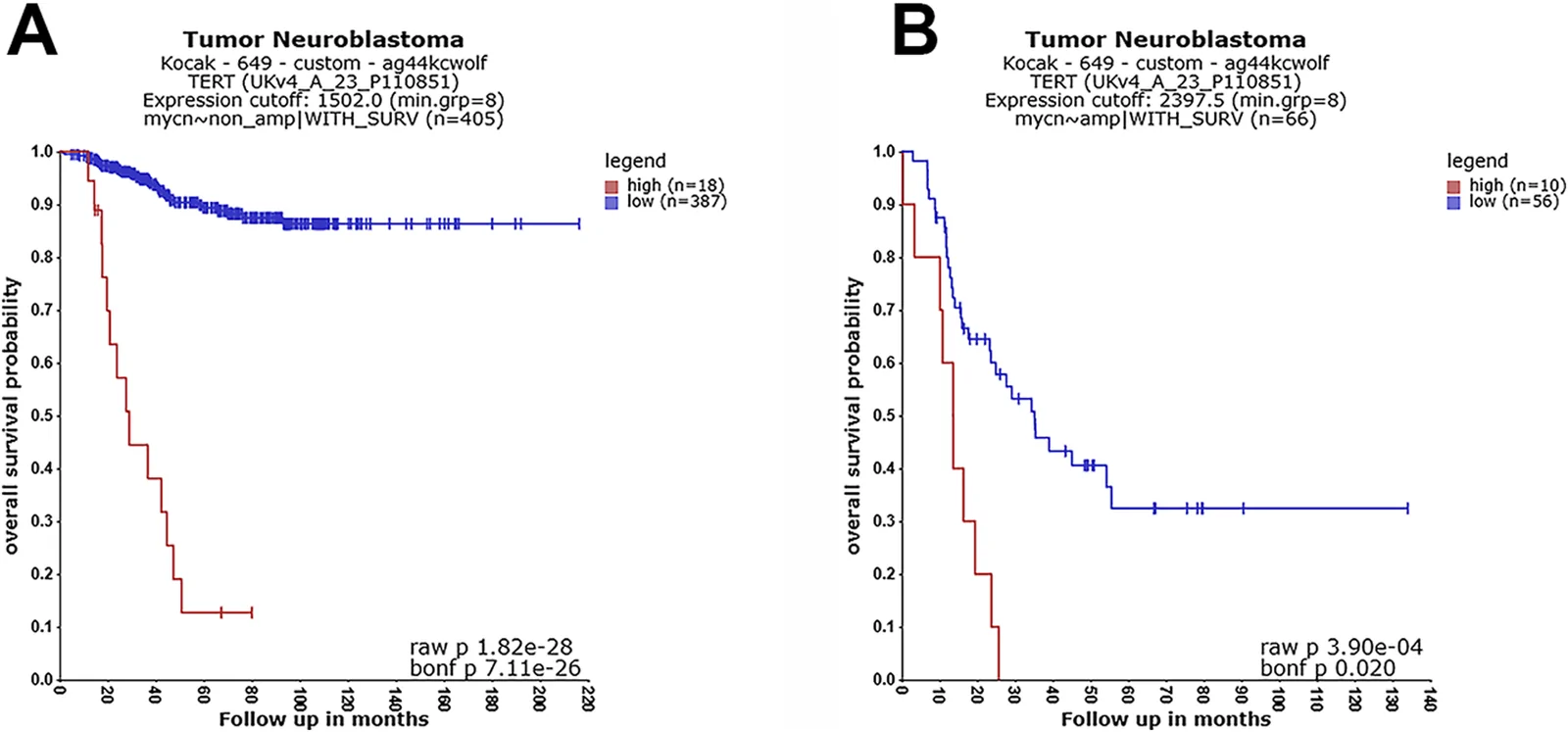
TERT expression and Kaplan–Meier survival analysis in neuroblastoma patients. **(A)** MYCN non amplified tumors and **(B)** MYCN amplified tumors from the Kocak dataset (R2 Genomics) were stratified by TERT expression. Red indicates high TERT expression and blue indicates low TERT expression.

To understand the clinical relevance of hTERT in neuroblastoma, we analyzed the Kocak dataset on *R* ^2^ Genomics. Amplification of MYCN gene is a major driving factor of neuroblastoma. Therefore, tumors were stratified based on MYCN amplification status and TERT expression levels. High TERT expression was observed in a subset of both MYCN amplified and non amplified tumors. Kaplan–Meier survival analysis revealed that patients with tumors expressing high levels of TERT had significantly poorer overall survival compared with those with low TERT expression, independent of MYCN amplification (Figure 3). This suggests that elevated TERT may contribute to aggressive tumor behavior beyond MYCN status.

These findings provided a rationale for selecting neuroblastoma cell lines with active telomerase for *in vitro* experiments, as hTERT targeted therapies, such as MK0873 and BIBR1532, are expected to have the greatest impact in cells with higher telomerase activity.

### 3.4 MK0873 directly reduces telomerase activity in a TRAP assay

**FIGURE 4:**
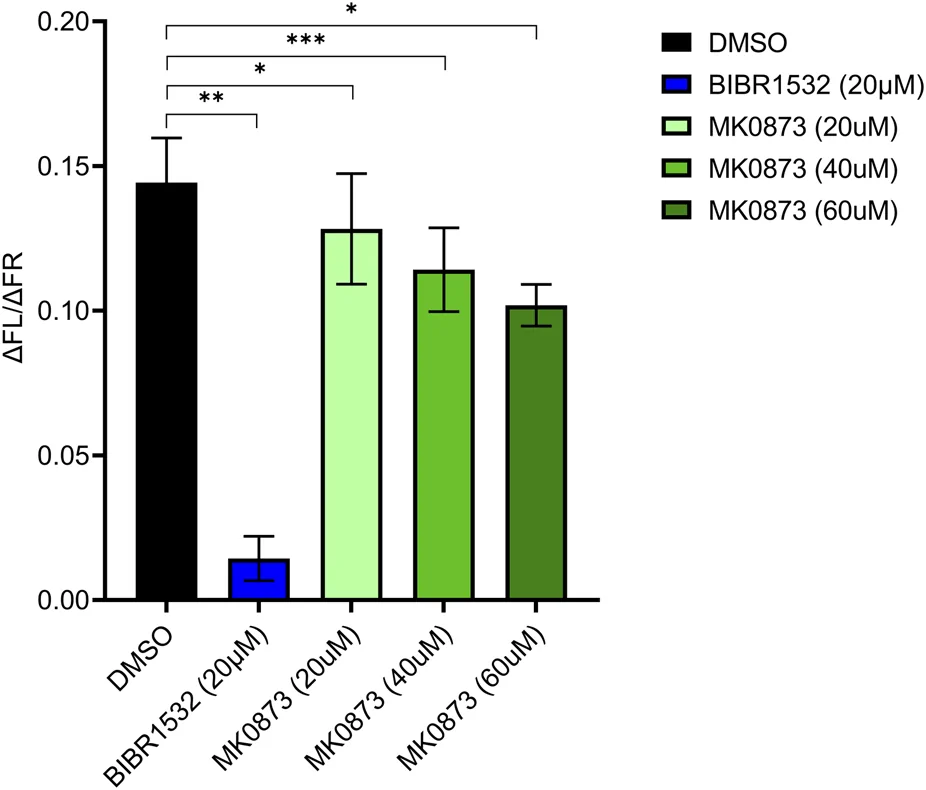
MK0873 inhibits telomerase activity in a concentration dependent manner in a cell free TRAP assay. Telomerase activity was measured in Jurkat cell lysates (30 ng protein per reaction). MK0873 (20, 40 and 60 µM), BIBR1532 (20 µM), or DMSO was added directly to the TRAP reaction mixture prior to PCR amplification. Telomerase activity is presented as the background corrected ΔFL/ΔR ratio. Data are presented as mean ± SD from three independent biological experiments. Statistical analysis was performed using one way ANOVA followed by Dunnett’s multiple comparisons test. *P* < 0.05, P < 0.01, *P* < 0.001 versus the DMSO control.

To directly evaluate the effect of MK0873 on telomerase catalytic activity, a fluorescence based TRAP assay was performed using Jurkat cell lysates. Compared with the DMSO control, MK0873 significantly reduced telomerase activity at 20 µM (*P* = 0.0382), 40 µM (*P* = 0.0007) and 60 µM (*P* = 0.0270). A progressive reduction in telomerase activity was observed with increasing MK0873 concentrations over the tested range. As expected, the reference inhibitor BIBR1532 (20 µM) produced a greater reduction in telomerase activity than MK0873 (*P* = 0.0026) Figure 4.

**FIGURE 5:**
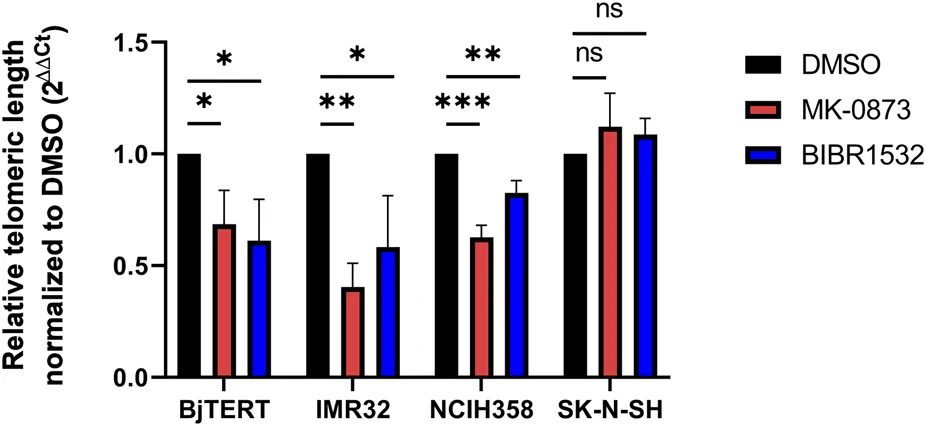
Relative telomere length measured by qPCR after 4 days of treatment with 10 µM MK0873 (Red) or BIBR1532 (Blue) compared to DMSO (Black) controls in BjTERT, IMR32, NCIH358 and SK-N-SH cells. Data are presented as mean ± SD of three repeats. Statistical significance was determined using one way ANOVA with Dunnett’s multiple comparisons test. Significance is indicated as *, ** and *** for p < 0.05, p < 0.01 and p < 0.001, respectively.

### 3.5 Telomere length quantification by qPCR

Telomere length was assessed by qPCR after 4 days of treatment with 10 µM MK0873 or BIBR1532, using DMSO treated cells as control and HBG as the single copy reference gene for normalization. The data is presented in Table 2.

**TABLE 2:** Relative telomere length after telomerase inhibitor treatment.

Cell line	MK0873			BIBR1532		
	Rep 1	Rep 2	Rep 3	Rep 1	Rep 2	Rep 3
BjTERT	0.555,233	0.851,285	0.650,068	0.402,104	0.676,459	0.755,923
IMR32	0.287,307	0.434,111	0.492,243	0.319,464	0.704,723	0.726,993
NCIH358	0.689,002	0.603,608	0.589,188	0.887,164	0.780,833	0.807,239
SK-N-SH	1.029337	1.294,306	1.043694	1.054896	1.169,308	1.035919

Relative telomere length was assessed by qPCR after 4 days of treatment with 10 µM MK0873 or BIBR1532 compared to DMSO controls, the data is presented in Figure 5. One way ANOVA followed by Dunnett’s multiple comparisons test revealed significant treatment effects across multiple cell lines.

In IMR32, MK0873 caused the most pronounced reduction (0.41 ± 0.06, *p* < 0.01), stronger than BIBR1532 (0.58 ± 0.13, *p* < 0.05). Both compounds significantly shortened telomeres in BjTERT (MK0873: 0.69 ± 0.09, BIBR1532: 0.61 ± 0.11, *p* < 0.05) and NCIH358 (MK0873: 0.63 ± 0.03, BIBR1532: 0.83 ± 0.03, *p* < 0.05). In SK-N-BE (2), moderate reductions were observed for both MK0873 (0.84 ± 0.14) and BIBR1532 (0.79 ± 0.10, *p* < 0.05). No significant change was detected in SK-N-SH (MK0873: 1.12 ± 0.09, BIBR1532: 1.09 ± 0.04, ns). Collectively, MK0873 demonstrated broader and more consistent telomere shortening across hTERT positive lines, with IMR32 showing the strongest sensitivity.

### 3.6 Effect of MK0873 and BIBR1532 on cell growth

The effects of MK0873 and BIBR1532 on cell growth were evaluated in 5 cell lines (IMR32, SK-N-SH, NCIH358, SK-N-BE (2) and BjTERT). Cells were treated with DMSO (vehicle control), MK0873 (5µM and 10 µM), or BIBR1532 (5µM and 10 µM) and growth was monitored at multiple time points. Fold changes are reported relative to time point 0 h.

**FIGURE 6:**
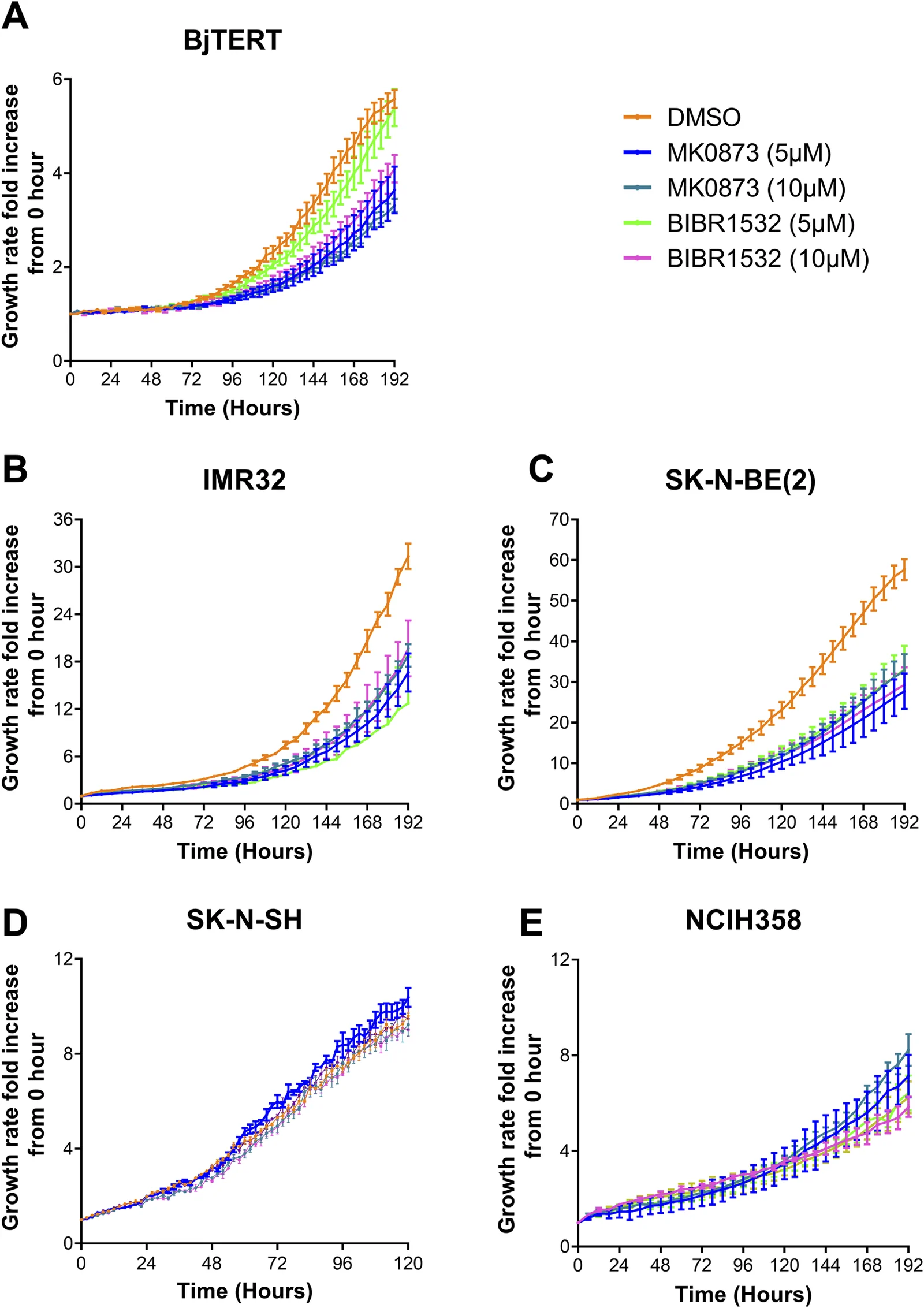
Proliferation of **(A)** BjTERT **(B)** IMR32, **(C)** SKNBE(2) **(D)** SK-N-SH and **(E)** NCIH358. Cells treated with MK0873 and BIBR1532 over the indicated time courses. Cells were treated with DMSO (orange), MK0873 at 5 µM (blue), MK0873 at 10 µM (cyan), BIBR1532 at 5 µM (green) and BIBR1532 at 10 µM (magenta). Cell growth was monitored continuously by live cell imaging and normalized to baseline (t = 0 h). Data represent mean ± SD of three replicates.

In BjTERT cells, DMSO controls showed a ∼5–6 fold increase in growth over 192 h. MK0873 markedly suppressed proliferation at both 5 µM (∼2.68 fold; ∼0.53 relative to DMSO) and 10 µM (∼3.04 fold; ∼0.61 relative to DMSO). In contrast, BIBR1532 showed little inhibition at 5 µM (∼4.79 fold; ∼0.95 relative to DMSO) but reduced growth more clearly at 10 µM (∼3.58 fold; ∼0.71 relative to DMSO), as depicted in Figure 6A.

In IMR32 cells, DMSO treated controls showed steady proliferation, reaching 31.98 ± 2.95 at 192 h (baseline, 1.0 fold). Both MK0873 and BIBR1532 significantly reduced cell growth at 5 μM and 10 µM. MK-0873 inhibited proliferation to 16.63 ± 5.57 (∼0.52 fold) at 5 μM and 18.12 ± 2.57 (∼0.57 fold) at 10 μM. BIBR1532 also suppressed proliferation, with 5 µM reducing growth to 11.20 ± 3.50 (∼0.35 fold) and 10 μM to 19.00 ± 4.42 (∼0.59 fold). Thus, both drugs were active in IMR32 cells at both concentrations, with MK0873 showing consistent inhibition and BIBR1532 demonstrating stronger suppression at the lower dose (Figure 6B).

In SK-N-BE (2) cells, DMSO treated controls reached 62.64 ± 0.76 at 192 h (1.0 fold). Both MK0873 and BIBR1532 significantly reduced proliferation at 5 μM and 10 µM. MK-0873 inhibited growth with 5 μM at 30.96 ± 0.79 (∼0.49 fold) and 10 μM at 39.20 ± 0.88 (∼0.63 fold), while BIBR1532 suppressed proliferation with 5 μM at 29.15 ± 0.52 (∼0.47 fold) and 10 μM at 33.26 ± 0.63 (∼0.53 fold) as presented in Figure 6C.

Proliferation of SK-N-SH cells remained steady across all treatments over 120 h, with final values comparable to DMSO controls (fold changes ≈1.0). Neither MK0873 nor BIBR1532 caused meaningful changes in growth and no clear dose dependent effects were observed (Figure 6D).

In NCIH358 cells, proliferation remained similar across all conditions over 192 h. Fold changes relative to DMSO were close to 1.0, indicating minimal effect of either compound under the tested conditions (Figure 6E).

Overall, MK0873 consistently inhibited growth across hTERT positive cell lines, showing robust activity at both 5 μM and 10 µM without clear dose dependence. In contrast, BIBR1532 displayed a more variable response, strongly suppressing proliferation in IMR32 and SK-N-BE (2) cells at both concentrations but exerting significant effects in BjTERT cells only at the higher dose. SK-N-SH (ALT-positive) and NCI-H358 (p53 deficient) cells were largely insensitive, with fold changes remaining close to 1.0.

### 3.7 X-gal senescence assay

**FIGURE 7:**
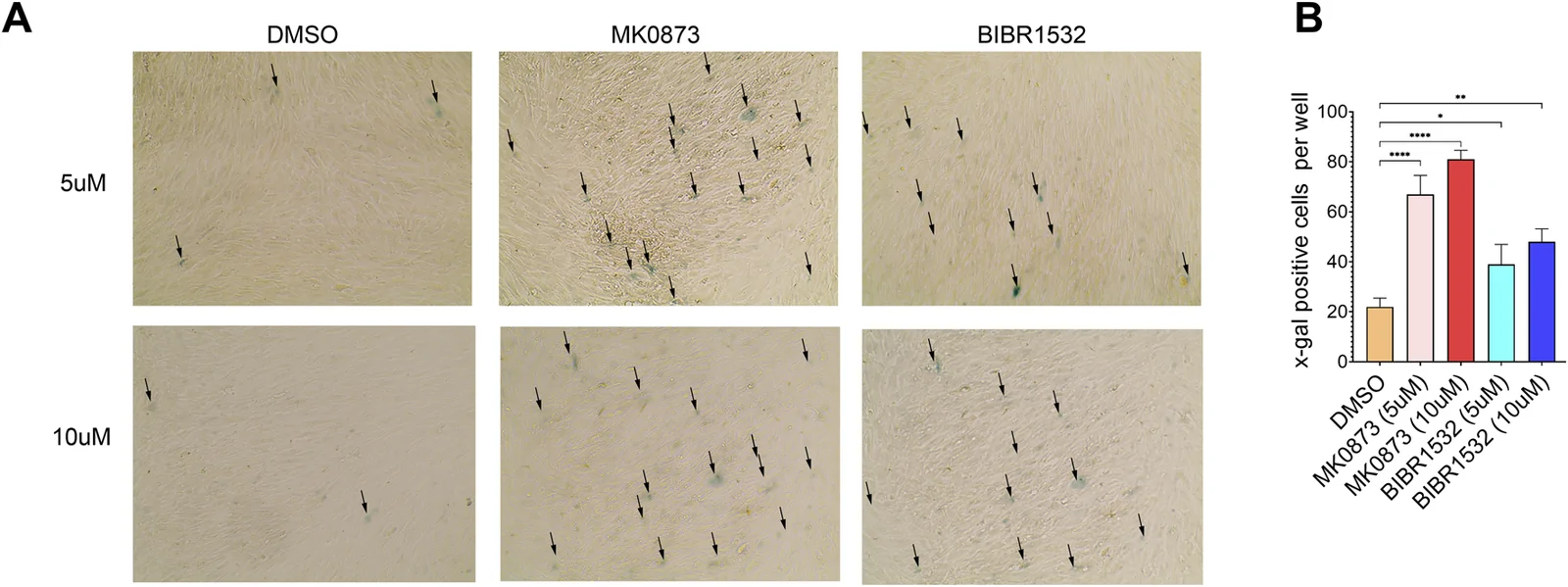
MK0873 induces stronger cellular senescence than BIBR1532 in BjTERT cells. **(A)** Representative images of BjTERT cells after 6 days of treatment and SA-β-gal staining. Cells were treated with DMSO (left), MK0873 (5 µM middle), or BIBR1532 (right) Upper row represents treatment with 5 µM while lower row 10 µM treatment. Senescent cells are indicated with black arrows and appear flat with prominent blue staining, reflecting β-galactosidase activity. MK0873 treatment resulted in more extensive senescence than BIBR1532. **(B)** Quantification of senescent cells per well (mean ± SD, n = 3). BjTERT cells treated with MK0873 showed the highest number of senescent cells at both concentrations, while BIBR1532 induced moderate senescence relative to DMSO controls. Color coding in the graph: orange = DMSO, pink = MK0873 (5 µM), red = MK0873 (10 µM), cyan = BIBR1532 (5 µM), blue = BIBR1532 (10 µM). Statistical significance is indicated as *, **, *** and **** for p < 0.05, p < 0.01, p < 0.001 and p < 0.0001, respectively.

hTERT inhibition is expected to trigger senescence, we next assessed the effect of MK0873 and BIBR1532 in BjTERT. For this purpose BjTERT cells were seeded (∼2,500 cells per well) in 96 well plates and treated with MK0873 (5 and 10 µM), BIBR1532 (5 and 10 µM), or DMSO (control). After 6 days, SA-β-gal staining was performed and senescent (blue stained) cells were manually counted as shown in Table 3 and depicted in Figures 7A,B.

**TABLE 3:** Number of X-gal positive BjTERT cells after treatment with MK0873 and BIBR1532.

Repeat #	DMSO	MK0873		BIBR1532	
		5 µM	10 µM	5 µM	10 µM
1	18	66	80	30	51
2	24	75	85	45	51
3	24	60	78	42	24

the stronger effect. One way ANOVA, confirmed a significant effect of treatment compared with DMSO (p < 0.0001). Multiple comparisons indicated that MK0873 induced significantly higher senescence than BIBR1532 at both tested concentrations. The effect of MK0873 was clearly dose dependent.

DMSO treated control wells displayed only a small number of senescent cells (18–24 per well), consistent with the low baseline level of spontaneous senescence. In contrast, MK0873 markedly increased senescence in a concentration dependent manner, with 60–75 blue cells at 5µM and 78–85 at 10 µM BIBR1532 also induced senescence, though less strongly than MK0873, with 30–45 blue cells at 5µM and 42–51 cells at 10 µM.

Morphologically, drug treated cells showed classic features of senescence, including enlarged, flattened shapes and intense cytoplasmic blue staining as shown in arrow in. These results indicate that both MK0873 and BIBR1532 promote senescence in BjTERT cells, with MK0873 showing.

## 4 Discussion

Telomerase, despite being reactivated in most of cancers, remains a challenging therapeutic target due to its structural complexity, slow acting biology and the difficulty of achieving target specificity. In normal somatic cells, telomere elongation is largely absent, making hTERT inhibition an attractive strategy for cancer therapy (Chen et al., 2020). However, the gap between preclinical promise and clinical translation has remained substantial. Drug repurposing provides a pragmatic strategy to accelerate therapeutic development by leveraging the established pharmacokinetic, safety and clinical data of existing compounds, thereby reducing the time and cost associated with *de novo* drug discovery.

BIBR1532, a benchmark small molecule telomerase inhibitor, binds the FVYL motif in the thumb domain of TERT, causing progressive telomere shortening and eventual cellular senescence (El-Daly et al., 2005; Damm et al., 2001; Pascolo et al., 2002). Structural insights into BIBR1532 action are primarily derived from its complex with *Tribolium castaneum* TERT (tcTERT). Homology modeling suggests that BIBR1532 may bind to the same region, known as the FYVL domain in human TERT (Bryan et al., 2015). BIBR1532 contains an α,β-unsaturated carbonyl group that functions as a Michael acceptor, enabling covalent interactions with nucleophilic residues of hTERT. Although it effectively inhibits telomerase, its physicochemical properties and limited clinical development have restricted its translational potential.

In contrast, MK0873 offers several features that support its further investigation. Molecular modelling predicts stable binding within the FVYL region of hTERT through non covalent interactions, including hydrogen bonding with Thr1098, avoiding irreversible modification. Its favorable physicochemical profile (Log P = 2.31 vs. 4.58 for BIBR1532; Caco-2 permeability = 1.001 vs. 0.456) suggests improved solubility, cellular uptake and safety potential. Importantly, the prior clinical evaluation of MK0873 as a PDE4 inhibitor provides pharmacokinetic and safety information that is unavailable for most experimental telomerase inhibitors, making it an attractive starting point for drug repurposing. (Pauwels et al., 2001; Crowley and Gooderham, 2023). Although a Phase II COPD trial was terminated (NCT00132730).

An important consideration in targeted drug discovery is that biochemical potency does not always predict cellular efficacy (Mateus et al., 2017). In the present study, MK0873 inhibited telomerase activity in the TRAP assay, although to a lesser extent than BIBR1532. Nevertheless, MK0873 produced comparable biological effects in several cellular models. One possible explanation is that its favorable physicochemical properties improve intracellular exposure, allowing sustained target modulation despite relatively modest inhibition in a cell free system. This interpretation remains speculative and requires experimental validation, but it highlights that compound permeability, intracellular retention and pharmacokinetic behavior can be as important as intrinsic enzyme potency when evaluating lead compounds.

The biological activity of MK0873 also appeared to depend on the mechanism of telomere maintenance. Telomerase dependent models were sensitive to treatment, whereas ALT positive cells showed little response, consistent with the expectation that inhibition of hTERT primarily affects cancers that rely on telomerase for telomere maintenance. Likewise, the continued proliferation of p53 deficient cells despite telomere shortening is consistent with the established role of p53 in mediating cell cycle arrest following telomere dysfunction (Chin et al., 1999). These observations reinforce the concept that both telomere maintenance mechanism and p53 status are likely to influence responses to telomerase targeted therapies and should be considered during patient stratification. These findings are consistent with recent reports demonstrating that rationally designed small molecule anticancer agents suppress tumor cell proliferation through selective molecular targeting, further supporting the utility of mechanism based approaches for anticancer drug development (Ewieda et al., 2025; Ewieda et al., 2026).

The reduction in relative telomere length after 4 days of treatment should be interpreted with caution, because progressive telomere shortening is generally considered a cumulative process that requires multiple population doublings under sustained telomerase inhibition (Chen et al., 2002). Classic telomerase inhibition studies in human tumor cells show that robust telomere erosion and growth arrest typically emerge only after weeks of continuous inhibition. Thus, the early changes observed here may instead reflect telomere dysfunction, replication associated stress, or rapid activation of ATM/ATR dependent DNA damage signalling at chromosome ends, as reported upon telomere uncapping, shelterin disruption, or oxidative/replication stress at telomeres (Delhommeau et al., 2002; Jullien et al., 2013). Discriminating between cumulative telomere erosion and these early damage related mechanisms will require longer treatment periods and additional mechanistic assays (Chin et al., 1999; Harman et al., 2024; Coluzzi et al., 2019). Although the computational, biochemical and cellular findings collectively support hTERT as a relevant molecular target, direct target engagement in living cells has not yet been demonstrated. In addition, because MK0873 was originally developed as a selective PDE4 inhibitor, the contribution of PDE4 inhibition to the observed cellular phenotype cannot currently be excluded. Addressing these questions through cellular target engagement studies, inducible PDE4 loss of function models and structure guided optimization of MK0873 analogues will be important for establishing target specificity and improving hTERT selectivity.

Overall, the present study identifies MK0873 as a promising non covalent scaffold for telomerase targeted drug discovery. Rather than establishing MK0873 as a fully optimized telomerase inhibitor, these findings provide proof of concept evidence that clinically characterized molecules can be successfully repurposed to target hTERT. The combination of favorable pharmacological properties, prior clinical experience and measurable biochemical and cellular activity provides a strong foundation for future medicinal chemistry optimization and translational development.

## Data Availability

The datasets presented in this study can be found in online repositories. The names of the repository/repositories and accession number(s) can be found in the article/Supplementary Material.
