# Supplementary material for: Repurposing MK0873 as a non-covalent hTERT-Targeting compound: a proof of concept study
Source: Front Aging. 2026 Aug 26;7:1783019. doi: 10.3389/fragi.2026.1783019 (PMC13553947; doi:10.3389/fragi.2026.1783019)
Supplement: Supplementary file 2 [file Table2.docx]

**Supplementary Methods**

**S1. Virtual Screening Protocol**

The DrugBank ligand library [1] was downloaded in .sdf format and preprocessed using OpenBabel [2] by adding hydrogens and generating 3D structures. Ligands were converted into .pdbqt format using PyRx [3] for AutoDock Vina, while the processed .sdf file was used for GOLD (CCDC). The hTERT structure (PDB ID: 7BG9, 3.80 Å) was obtained from the RCSB PDB, with water molecules, ligands and non-essential ions removed; missing hydrogens were added, Kollman charges assigned and the protein prepared in formats compatible with both docking platforms. The receptor grid was centered on the FVYL motif (F1012, V1025, Y1089, L1092), the binding site of BIBR1532 [4], at coordinates X:116.124, Y:137.513, Z:108.558. High-throughput screening was performed with GPU-accelerated AutoDock Vina [5].

**S2. MM/GBSA calculations**

To refine the binding free energy of the top 200 molecules screened with AutoDock Vina, we performed MM/GBSA calculations using the Uni-GBSA toolkit [6]. MM/GBSA combines molecular mechanics and solvation energy components, providing more accurate post-docking evaluation. Protein–ligand complexes obtained from AutoDock Vina were used as input, with system topologies generated and implicit solvation modeled using the Generalized Born (GB) approach. Standard configurations were applied to re-prioritize ligands for further analysis. To eliminate steric clashes and optimize local geometry, the complexes were subjected to brief energy minimization using the gaff force field. The binding free energy was calculated as:

$$\Delta G\text{Total}=\text{ }\text{G}\text{complex}-\text{(}\text{G}\text{protein}\text{​+}\text{G}\text{ligand}\text{​}\text{)}$$

Where G is the estimated free energy (molecular mechanics + solvation + surface area). To normalize binding affinity with respect to molecular size, ligand efficiency (LE) was calculated as:

$\text{LE}\text{=}\frac{\text{ΔG}\text{Total}}{\text{N}\text{heavy}}$

Where ΔG_Total_ is the MM/GBSA binding energy (kcal/mol), N_heavy_ is the number of non hydrogen heavy atoms in the ligand.

**S3. Molecular dynamics simulation**

Molecular dynamics (MD) simulations of the lead complexes were performed using GROMACS 2023.1 [7]. Each complex was placed in a triclinic periodic boundary box, solvated with the TIP3P water model (buffer distance 1.5nm) and neutralized to 0.15 M NaCl using the Genion tool. Energy minimization was performed using the steepest descent algorithm to a maximum force of 1000 kJ/mol/nm. The system was equilibrated in two phases: NVT (100 ps, 300 K, V-rescale thermostat) and NPT (100 ps, 1 bar, Parrinello–Rahman barostat). Production MD was first run for 400 ns, followed by a second 100 ns run starting from the final frame of the first trajectory, resulting in a total of 500 ns simulation. Simulations employed a 2-fs timestep with periodic boundary conditions, Particle Mesh Ewald (PME) for electrostatics and bond constraints. Trajectories were saved every 10 ps and analyzed for RMSD and hydrogen-bond patterns using GROMACS tools (gmx rms and gmx hbond), with visualization done in VMD [8].

**S4. Telomere Length Measurement Assay**

After treatment cells were harvested, genomic DNA was extracted using QIAamp DNA extraction kit (Qiagen, #56304) following the manufacturer protocol. DNA was quantified using Nanodrop (Thermo Scientific 2000C) and 50 ng of DNA per sample was used for qPCR. Human β-globin (HBG) served as the single-copy reference gene. Primers were purchased from Life Technologies, all the primers and their concentrations used in current study are previously validated [9, 10]. Sequences of primers are as follows:

Telomere Forward: 5′-CGGTTTGTTTGGGTTTGGGTTTGGGTTTGGGTTTGGGTT-3′

Telomere Reverse: 5′-GGCTTGCCTTACCCTTACCCTTACCCTTACCCTTACCCT-3′

HBG Forward: 5′-GCTTCTGACACAACTGTGTTCACTAGC-3′

HBG Reverse: 5′-CACCAACTTCATCCACGTTCACC-3′

qPCR reactions (10 μL) were prepared as given in Table 1:

Table 1: Recipe for qPCR reaction mixture

| **Reagent** | **Volume/Reaction (μL)** |
| --- | --- |
| DNA (50 ng) | 1 |
| 2X Fast SYBR® Green Master Mix  (Applied Biosystems, # 4472908) | 5 |
| Telomere Forward (1 μM) or HBG Forward (3 μM) | 1 |
| Telomere Reverse (3 μM) or HBG Reverse (7 μM) | 1 |
| Nuclease-free water | 2 |
| Total | 10 |

StepOne™ Real-Time PCR Systems (Applied Biosystem) were used for thermocyclic reaction. And relative telomeric length was calculated by 2∆∆Ct method. Conditions of qPCR are as follows:

Table 2: Step wise conditions for PCR

|  | **Hold** | **Amplification** | | **Melting Curve** | | |
| --- | --- | --- | --- | --- | --- | --- |
|  |  | Melting | Annealing |  |  |  |
| Telomere primers | 95 ℃  3 min | 95 ℃  15 Sec | 56 ℃  1 min | 95 ℃  15 Sec | 56 ℃  1 min | 95 ℃  15 sec |
| β-globin primers | 95 ℃  3 min | 95 ℃  15 Sec | 58 ℃  1 min | 95 ℃  15 Sec | 58 ℃  1 min | 95 ℃  15 sec |

1.	Knox, C., et al., *DrugBank 6.0: the DrugBank Knowledgebase for 2024.* Nucleic Acids Res, 2024. **52**(D1): p. D1265-D1275.

2.	O'Boyle, N.M., et al., *Open Babel: An open chemical toolbox.* J Cheminform, 2011. **3**: p. 33.

3.	Dallakyan, S. and A.J. Olson, *Small-molecule library screening by docking with PyRx.* Methods Mol Biol, 2015. **1263**: p. 243-50.

4.	Steczkiewicz, K., et al., *Human telomerase model shows the role of the TEN domain in advancing the double helix for the next polymerization step.* Proceedings of the National Academy of Sciences, 2011. **108**(23): p. 9443-9448.

5.	Tang, S., et al., *Accelerating AutoDock Vina with GPUs.* Molecules, 2022. **27**(9).

6.	Yang, M., et al., *Uni-GBSA: an open-source and web-based automatic workflow to perform MM/GB(PB)SA calculations for virtual screening.* Briefings in Bioinformatics, 2023. **24**(4).

7.	Abraham, M.J., et al., *GROMACS: High performance molecular simulations through multi-level parallelism from laptops to supercomputers.* SoftwareX, 2015. **1-2**: p. 19-25.

8.	Humphrey, W., A. Dalke, and K. Schulten, *VMD: visual molecular dynamics.* J Mol Graph, 1996. **14**(1): p. 33-8, 27-8.

9.	Ksiazek, K., et al., *Premature senescence of mesothelial cells is associated with non-telomeric DNA damage.* Biochem Biophys Res Commun, 2007. **362**(3): p. 707-11.

10.	Joglekar, M.V., et al., *An Optimised Step-by-Step Protocol for Measuring Relative Telomere Length.* Methods Protoc, 2020. **3**(2).
